# Racial discrimination in medical care settings and opioid pain reliever misuse in a U.S. cohort: 1992 to 2015

**DOI:** 10.1371/journal.pone.0226490

**Published:** 2019-12-20

**Authors:** Samuel L. Swift, M. Maria Glymour, Tali Elfassy, Cora Lewis, Catarina I. Kiefe, Stephen Sidney, Sebastian Calonico, Daniel Feaster, Zinzi Bailey, Adina Zeki Al Hazzouri

**Affiliations:** 1 Center for Health Equity in Kidney Disease, University of New Mexico School of Medicine, Albuquerque, NM, United States of America; 2 Department of Epidemiology and Biostatistics, University of California San Francisco, San Francisco, CA, United States of America; 3 Division of Epidemiology, Department of Public Health Sciences, University of Miami, Miami, FL, United States of America; 4 Division of Preventative Medicine, Department of Medicine, University of Alabama, Tuscaloosa, AL, United States of America; 5 Department of Quantitative Health Sciences, University of Massachusetts Medical School, Boston, MA, United States of America; 6 Division of Research, Kaiser Permanente Northern California, Oakland, CA, United States of America; 7 Department of Health Policy and Management, Columbia University, New York, NY, United States of America; 8 Division of Biostatistics, Department of Public Health Sciences, University of Miami, Miami, FL, United States of America; 9 Jay Weiss Institute for Health Equity, Sylvester Comprehensive Cancer Center, University of Miami, Miami, FL, United States of America; 10 Department of Epidemiology, Mailman School of Public Health, Columbia University, New York, NY, United States of America; University of Maryland, UNITED STATES

## Abstract

**Background:**

In the United States whites are more likely to misuse opioid pain relievers (OPRs) than blacks, and blacks are less likely to be prescribed OPRs than whites. Our objective is to determine whether racial discrimination in medical settings is protective for blacks against OPR misuse, thus mediating the black-white disparities in OPR misuse.

**Methods:**

We used data from 3528 black and white adults in the Coronary Artery Risk Development in Young Adults (CARDIA) study, an ongoing multi-site cohort. We employ causal mediation methods, with race (black vs white) as the exposure, lifetime discrimination in medical settings prior to year 2000 as the mediator, and OPR misuse after 2000 as the outcome.

**Results:**

We found black participants were more likely to report discrimination in a medical setting (20.3% vs 0.9%) and less likely to report OPR misuse (5.8% vs 8.0%, OR = 0.71, 95% CI = 0.55, 0.93, adjusted for covariates). Our mediation models suggest that when everyone is *not discriminated* against, the disparity is wider with black persons having even lower odds of reporting OPR misuse (OR = 0.63, 95% CI = 0.45, 0.89) compared to their white counterparts, suggesting racial discrimination in medical settings is a risk factor for OPR misuse rather than protective.

**Conclusions:**

These results suggest that racial discrimination in a medical setting is a risk factor for OPR misuse rather than being protective, and thus could not explain the seen black-white disparity in OPR misuse.

## Introduction

Drug overdose death rates within the United States nearly tripled between 1999 and 2015[1]. Vital records data, opioid sales data, and other sources suggests that a large portion of this increase in deaths is attributable to legally produced, pharmaceutical Opioid Pain Relievers (OPRs) such as oxycodone, hydromorphone and oxymorphone[2, 3]. The National Institute on Drug Abuse (NIDA) defines misuse of prescription drugs as “taking a medication in a manner or dose other than prescribed; taking someone else’s prescription, even if for a legitimate medical complaint such as pain; or taking a medication to feel euphoria (i.e., to get high)”[4].

During the prescription opioid epidemic, death rates attributed to OPR overdose among whites have been more than twice that of the rates seen among blacks[3], and whites have had significantly higher rates of hospitalization due to OPRs than blacks[5]. The black and white disparity in drug related overdose deaths in the 2000s was drastically different than it was in the 1990s[1]. According to CDC mortality files, in 1999 blacks were more likely to overdose from drug induced causes than whites, however around 2002 the rate for whites overtook the rate for blacks[1, 6], and by 2015 mortality rates for whites were approximately 1.5 times higher than blacks[1]. From the 1990s to early 2010s OPR misuse was attributable mostly or in part to patterns of OPR overprescribing in the United States[2]. The sales of OPRs within the United States increased by almost 500% between 1999 and 2011 and the same time the rates of OPR related deaths were tripling[2]. Given this knowledge, it would follow that any racial/ethnic disparities in OPR misuse could be related to disparities in OPR prescribing, based on the premise that less prescribing for minorities might result in fewer opportunities for misuse. A recent review paper found that across 70 studies of prescribing patterns, racial and ethnic minorities received less treatment for acute and chronic pain than their white counterparts[7]. A study of disparities in opioid prescribing in pain-related emergency room visits also showed blacks to have a lower odds of receiving OPRs than whites[8]. These disparities pose several interesting questions related to discrimination in medical care and drug misuse in the United States. Perceived interpersonal discrimination (henceforth called discrimination) due to race within the medical care setting has been associated with negative healthcare service outcomes and underutilization of preventive services[9, 10]. Using an experimental design and actors playing cardiac patients, one study found that given identical clinical presentations for white and black patients, physicians were more likely to recommend white patients for cardiac catheterization than black patients[11]. In another study, Krieger et al. hypothesized that the lack of prescription of hormone therapy to black women, due to discrimination, was likely the reason why black women had lower rates of breast cancer in the 1990s before the results of the Women’s Health Initiative Study showed an association between these hormones and breast cancer[12]. With these examples in mind, we propose to investigate a research question in which discrimination in a healthcare setting may actually be protective against negative consequence of access to healthcare.

The objective of this study is to examine the relationship between race and OPR misuse (i.e. the black-white disparity), and whether discrimination in a medical setting acts as a mediator in this relationship, employing causal mediation framework. Following the evidence from the studies noted above[11, 12], we hypothesized that racial discrimination in a medical setting would result in under prescribing of OPR to blacks, which would then result in lower OPR misuse among blacks and thus explain the black-white disparity.

## Methods

### Study population

CARDIA is an ongoing multisite prospective cohort study of the determinants of clinical and subclinical cardiovascular disease. In 1985 and 1986 a total of 5,114 black and white adults were recruited into this study from four field centers which include: The University of Alabama at Birmingham, The University of Minnesota, Northwestern University, and Kaiser Permanente in Oakland California. Participants were followed from 1985–86 to the present with follow up visits in 1987–88, 1990–91, 1992–93, 1995–96, 2000–01, 2005–06, 2010–11, and 2015–16. Details of the study design are described elsewhere[13]. The CARDIA study was IRB approved at each field center, and written informed consent was obtained from each participant at each examination. This secondary analysis of de-identified data was approved by the CARDIA Publications and Presentations Subcommittee. CARDIA data can be accessed after a manuscript proposal and request for data acquisition are approved by the appropriate Presentations and Publications (P&P) committee.

### Perceived discrimination in a medical setting prior to year 2000

Discrimination is measured using the Experiences of Discrimination (EOD) instrument, which has been previously validated[14]. The discrimination questions were asked in years 1992, 2000, 2005, 2010 and 2015. Study participants were asked “have you *ever* experienced discrimination, been prevented from doing something, or been hassled, or made to feel inferior in any if the following situations because of *your race*?”. They were then presented with situations/domains, which included, “at school,” “getting a job,” “getting housing,” “at work,” “at home,” “getting medical care,” or “on the street in a public setting”. In this study, we used racial discrimination in “getting care in a medical setting” (and *not* in any other setting). We used discrimination from exam years 1992 and 2000 for our exposure of interest, thus reflecting any lifetime reporting of discrimination up to year 2000. As such, we did not exclude those who have changed their report of discrimination across the years. We chose to examine discrimination up to year 2000 to examine the influence of prior discrimination on the largest increases in the black-white disparity in OPR misuse, which happened after the year 2000. These two exam years were combined to increase the sample size of persons who experienced discrimination in a medical setting, while we used other approaches/definitions in sensitivity analyses (described later).

### Nonmedical Opioid Pain Reliever (OPR) misuse from 2005 to 2015

CARDIA participants were asked about *lifetime* illicit drug misuse in all examination years. For this analysis, we combined lifetime OPR misuse in exam years 2005, 2010 and 2015 as our outcome, excluding people who reported OPR or opiate misuse in years 2000 and prior. This allowed us to capture new misusers and that OPR misuse took place after our exposure period (up to 2000) (defined above). Participants were asked questions related to misuse of illicit opioids such as “Heroin,” and misuse of OPR medications such as “Dilaudid,” “Morphine,” “Demerol,” “Oxycodone,” “Hydromorphone,” or “other prescription opioids” for non-medical reasons. In all exam years, participants who reported non-medical use of prescription OPR medications were included as OPR misusers, and participants who misused only heroin or other non-prescription illicit opioids were excluded from the OPR misuse group.

### Other covariates

Participants reported their race (white vs. black), sex, age, years of educational attainment, total household income (collected in 8 brackets and treated continuously using the midpoint dollar amount of bracket), and health insurance status (insured vs. not). Parental SES was operationalized as the highest level of education in years achieved by any parent or guardian. Depressive symptoms were measured using the Center for Epidemiological Studies depression scale (CES-D) instrument, which includes 20 items and ranges from 0–60[15]. All covariates were measured in year 1992 (study baseline, and when discrimination was first measured), except for depressive symptoms which was measured in year 1990.

### Statistical analysis

Of the 4617 participants who were present at any exam between 1992 and 2015, we excluded 264 participants without a measure of lifetime discrimination in 1992/2000. Since the OPR question is also a lifetime measure, we further excluded 318 participants who by exam year 2000 had misused OPR at least once in their lifetime, and further excluded 507 participants with missing OPR measures in 2005, 2010 and 2015. This left a final analytical sample of 3528 persons. As such, for this analysis, we established temporality between exposure and outcome by defining the exposure period as lifetime medical discrimination prior to year 2000, and the outcome period as any OPR misuse in years 2005 through 2015.

**Fig 1:**
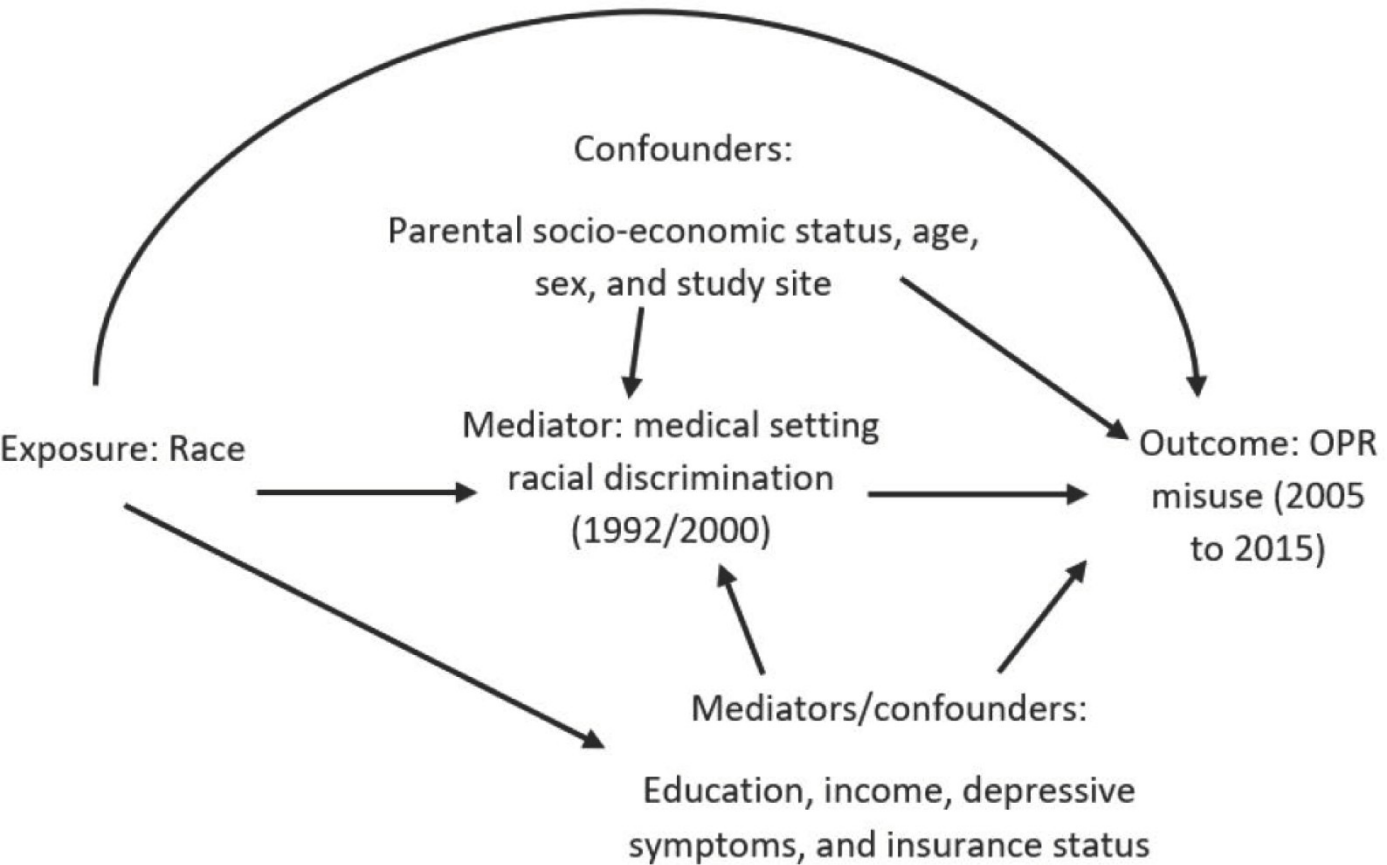
Directed acyclic graph for proposed relationship between race, discrimination in a medical care setting, and OPR misuse.

We first compared black and white participants across our covariate characteristics using chi-squared tests and t-tests. Next, we used a causal mediation framework to assess whether there is a racial disparity in OPR misuse, and if so, whether this disparity could be explained by discrimination. The directed acyclic graph (DAG) for this research question is presented in Fig 1. Race as the exposure/ treatment (white = 0 (reference), black = 1), discrimination in a medical care setting as the mediator (M = 1 discrimination, M = 0 no discrimination), and OPR misuse as the outcome (Y = 1 OPR misuse, Y = 0 no OPR misuse).

We performed causal mediation analysis as described by Valeri and VanderWheele[16], a method that uses counterfactual contrasts to compute a total effect (TE) and a controlled direct effect (CDE) for the relationship of interest. The total effect (TE) is the effect of race on OPR misuse, adjusted for measured confounders; and the controlled direct effect (CDE) is the effect of race on OPR misuse examining groups of participants who experience the same level of discrimination (either M = 0 or M = 1), adjusted for measured confounders. We focus on the CDE, comparing the influence of race among participants experiencing no discrimination (M set to 0). We consider the following variables as confounders of the discrimination–OPR path: parental SES, sex, age, and study site. Variables such as education, income, depressive symptoms, and insurance status are confounders of the discrimination–OPR path but are mediators of the race–OPR path (see Fig 1).

Since our outcome (OPR misuse) was rare (<10%), we used logistic regression[17] in constructing the following four models. In model 1, we calculate the total effect by regressing OPR misuse on race while adjusting for confounders (parental SES, age, sex, study site). Next, we calculate the controlled direct effect by comparing three models (models 2 to 4) all of which are regressing OPR misuse on race while adjusting for discrimination. In controlled direct effect model 2, we adjust for confounders only. In controlled direct effect model 3, we additionally adjust for the confounders/mediators. In controlled direct effect model 4, which is a marginal structural model, we used stabilized inverse probability weights (IPW) to account for the confounders/mediators (see supplemental material for the SAS code to create the stabilized treatment weights). The approach for these models is in line with previous mediation analysis literature[18]. We interpret the coefficient for race as describing the racial inequality in OPR misuse, and the controlled direct effect as the magnitude of the racial inequality we would expect if discrimination were absent for both blacks and whites.

We conducted three sensitivity analyses. First, we performed an analysis excluding participants who reported lifetime discrimination in 1992 but not in 2000, as this pattern while it could be true, it could also be reflecting measurement error in response to the question. Second, we conducted an analysis examining “recent” discrimination rather than lifetime discrimination, by including only persons who reported discrimination in a medical setting in 2000 and not in 1992 or prior. Third, since the opioid epidemic has been characterized by increases in prescribing of OPRs for various conditions related to both chronic and acute pain[2], we conducted an analysis adjusting for self-reported pain in year 2000 as a proxy variable for pain related conditions that may influence OPR misuse. Participants were asked the following question: “During the past 4 weeks, how much did pain interfere with your normal work (including work outside the home and housework)?”; and responses were coded as “no pain, a little bit of pain to moderate pain, or quite a bit of pain to extreme pain”. All analyses were conducted using SAS version 9.4 and SAS University Edition Software[19].

## Results

As shown in Table 1, black participants were much more likely to report experiencing discrimination in a medical setting (20.3% vs 0.9%) and less likely to report OPR misuse (5.8% vs 8.0%). Black participants were more likely to be female, have lower incomes, lower educational attainment, and lower parental SES, and were more likely to have elevated depressive symptoms (CES-D≥16).

**Table 1:** Baseline characteristics of study participants by race, CARDIA study, 1992–2015.

	Black n = 1717	White n = 1811	P value
Age (years), mean, (SD)	31 (3.8)	32 (3.4)	<0.01
Female, n, (%)	1040 (60.6)	998 (54.6)	<0.01
Years of education, mean, (SD)	13.84 (2.1)	15.6 (2.5)	<0.01
Household income ($), mean, (SD)	$32,027.5 ($18,492.4)	$43,633.9 ($19980.1)	<0.01
Parental years of education, mean, (SD)	12.79 (2.7)	14.92 (3.1)	<0.01
Had health insurance, n, (%)	1259 (73.3)	1456 (80.4)	<0.01
CARDIA study site, n, (%)			<0.01
Birmingham, n, (%)	469 (27.3)	375 (20.7)	
Chicago, n, (%)	355 (20.7)	427 (23.5)	
Minneapolis, n, (%)	339 (19.7)	571 (31.5)	
Oakland, n, (%)	554 (32.3)	438 (24.2)	
CES-D score ≥16, n, (%)	462 (26.9)	297 (16.4)	<0.01
Racial discrimination in a medical care setting, n, (%)	350, (20.4)	17 (0.9)	<0.01
OPR Misuse 2000–2015, n, (%)	100 (5.8)	145 (8.0)	<0.01

N is number of participants, SD is standard deviation. P values are from χ^2^ tests for categorical variables and t tests for continuous variables. CES-D: Center for Epidemiological Studies Depression Score, taken in 1990. All other covariates were measured in 1992; OPR: Opioid Pain Reliever, measured after 2000

Table 2 displays the results of the causal mediation analysis, in which the influence of discrimination can be understood by comparing the coefficients of the total effect and controlled direct effects. The total effect suggests that adjusting for all measured confounders, black persons have 0.71 times the odds of reporting OPR misuse (OR = 0.71, 95% CI = 0.55, 0.93) compared to their white counterparts, i.e., blacks are at an approximately 29% lower risk of OPR misuse than whites. Compared with the total effect, in all three controlled direct effect models the black-white disparity widens (coefficient gets further away from 1) suggesting that if no one were discriminated against, black persons would be at an even lower risk of OPR misuse compared to whites. Adjusting for all measured confounders, the controlled direct effect in model 2 suggests that when everyone is set to being *not discriminated* against (M = 0), black persons would have 0.63 times the odds of reporting OPR misuse (OR = 0.63, 95% CI = 0.45, 0.89) compared to their white counterparts, i.e. blacks would have a 37% lower risk of OPR misuse. Adjusting for all measured confounders as well as confounders/mediators, the controlled direct effect in model 3 suggests that when everyone is set to being *not discriminated* against (M = 0), black persons would have 0.55 times the odds (45% lower risk) of reporting OPR misuse (OR = 0.55, 95% CI = 0.38, 0.80) compared to their white counterparts. Finally, the controlled direct effect in model 4 which uses stabilized weights to account for measured confounding, suggests that when everyone is set to being *not discriminated* against (M = 0), black persons would have 0.65 times the odds (35% lower risk) of reporting OPR misuse (OR = 0.65, 95% CI = 0.46, 0.91) compared to their white counterparts.

**Table 2:** Relationship of race, discrimination in a medical setting, and OPR misuse using causal mediation methods, CARDIA study (N = 3528).

	Total effect (model 1)		CDE: Adjusted for discrimination and confounders (model 2)		CDE: Adjusted for discrimination, confounders, and confounders/mediators (model 3)		Marginal structural model (model 4)	
	OR	95% CI	OR	95% CI	OR	95% CI	OR	95% CI
Black vs. white	**0.71**	**(0.55, 0.93)**	**0.63**	**(0.45, 0.89)**	**0.55**	**(0.38, 0.80)**	**0.65**	**(0.46, 0.91)**
No discrimination vs. discrimination			**0.54**	**(0.34, 0.86)**	**0.50**	**(0.31, 0.83)**	**0.51**	**(0.31, 0.83)**

OR is odds ratio, CI is confidence interval, N is number of participants, CDE is controlled direct effect. Model 1 is adjusted for race; Model 2 is adjusted for race, medical discrimination and confounders (parental SES, age, sex, and study site); Model 3 is adjusted for race, medical discrimination, confounders (parental SES, age, sex, and study site), and confounders/mediators (education, income, depressive symptoms, and insurance status); Model 4 is adjusted for race and medical discrimination, and uses stabilized inverse probability weights to account for the confounders and confounders/mediators (see supplemental SAS code).

The results of our four sensitivity analyses can be seen in S1 Table, S2 Table, and S3 Table. In our first sensitivity analysis (S1 Table), we assessed measurement error in the lifetime discrimination measure by excluding 127 participants who inconsistently responded to the discrimination measure across years 1992 and 2000, and our results were largely similar to those seen in our main analysis (Table 2). In our second sensitivity analysis (S2 Table) including only “recent” experience of discrimination in a medical setting, our sample size of persons who experienced discrimination was drastically reduced (n = 116) and thus we were unable to draw conclusions from these models, however the point estimates were in the same direction as our main analysis. In our sensitivity analysis adjusting for self-reported pain as a confounder (S3 Table), the results were also similar to those seen in Table 2.

## Discussion

By comparing the total effects and controlled direct effects from our models, we show that discrimination in a medical setting increases a person’s risk of OPR misuse rather than being protective against OPR misuse. Our findings are counter to our original hypothesis that discrimination in a medical setting would be protective against iatrogenic (i.e. unintentionally harmful) effects of healthcare-related opioid misuse and thus a mediator of the black-white disparity. While our total effect of 0.71 suggests that blacks have lower odds of OPR misuse compared to whites–which is in line with the established rates of racial disparity in OPR misuse[3, 5]–in our final model our controlled direct effect of 0.65 became more protective (i.e. further away from one), thus widening the disparity and suggesting that discrimination in a medical setting is a risk factor, rather than a protective factor, for OPR misuse.

We hypothesized that racial discrimination in a medical setting would result in less treatment and thus under prescribing of OPR to blacks, which would then result in lower OPR misuse among blacks, and thus explain the observed black-white disparity. As our findings were counter to our hypothesis, we must consider the possibility that misused OPR medications originated from potential sources other than the medical setting, such as the diversion of OPR medications and black market sale of these medications. A recent paper estimated that 42% of OPRs prescribed in the emergency department may be ultimately misused, either by the patient or persons close to them[20]. Given these diversion rates, prescribing practices may have little impact on the actual availability of OPR medications for persons who would misuse them. Future studies using mediation analysis to examine the black-white disparity in OPR misuse, and with more detailed survey questions or criminal justice data, should account for the possible source of OPR medications. This could be a larger problem in the future, as recent research points to a new shift in the opioid epidemic since 2010, wherein the large disparity in black versus white OPR misuse from the 1990s to 2010 (largely driven by prescription OPRs) has narrowed in recent years due to the return to synthetic and illicit OPRs[6].

Another possible explanation for the findings related to the influence of discrimination is that the black—white disparity in OPR misuse is not a result of discrimination in medical settings, but other factors influencing racial differences in misuse of illicit opioids vs prescription OPR. While the focus of this paper is not illicit nonprescription opioids such as heroin, there were more blacks than whites who reported lifetime heroin misuse in the years after 2010 (6 whites vs 28 blacks reporting new lifetime heroin misuse after 2000). It is outside of the scope of these data to determine whether these differences in heroin misuse may be a result of other factors related to discrimination in medical care settings. Given the knowledge that there are known racial biases in OPR prescribing, it is possible that the lesser rate of OPR misuse among blacks could be offset by a higher rate of heroin misuse among blacks. While the black-white disparity in opioid misuse persists, other research suggests there are recent increases in illicit opioid misuse among blacks, which would be consistent with this alternate explanation[6, 21].

A third and related possible explanation for the findings is that there was not discriminatory prescribing practices influencing racial disparities in the opioid epidemic, but rather cautious prescribing to black persons to intentionally protect them from these drugs of potential abuse out of concern for the well-being of black patients. This explanation is plausible given the findings of this research, however, this explanation is not supported by the well documented history of discriminatory and unequal provision of care in OPR prescribing[7, 8] and healthcare as a whole[22]. Future studies could benefit from using qualitative and mixed methods approaches to identify alternative explanations for racial disparities in the opioid epidemic. Information from prescribers and drug counselors, black market contexts, as well as on the social histories of disparities of drug misuse would enrich our understanding of racial disparities in OPR misuse.

Our findings suggest that discrimination in a medical setting is a risk factor for OPR misuse. The relationship between discrimination in a medical care setting and OPR misuse we found is likely similar to the relationship observed between discrimination in any other setting and negative outcomes. In this cohort, experiences of discrimination in any setting have been associated with several negative health outcomes, including lower birthweight among mothers who reported such experiences[23], greater waist circumference[24], and increased sedentary behaviors[25]. There is also a large body of evidence supporting the relationship between perceived discrimination across all settings and increased substance misuse and risky behaviors among both whites, blacks and other groups[26–33], suggesting that substance misuse could be a coping mechanism for discrimination, regardless of the race or ethnicity. Further analysis could investigate similar mechanisms in the relationship between discrimination and OPR misuse. In a meta-analysis, discrimination in various settings was associated with worse mental health, physical health, heightened stress responses and importantly, participation in unhealthy behaviors[33]. Given that recent research has shown increases in black OPR overdose and shifting demographics[6, 21, 34], the results of this analysis may also be useful to inform prevention of OPR misuse among blacks. However, it is important to acknowledge that other factors may be stronger predictors of OPR misuse and therefore more important for informing prevention efforts.

Our study has several limitations. In CARDIA, we did not have an objective measure of OPR misuse such as by hair or urine testing. As a small number of whites reported being discriminated against in a medical setting (n = 17), this may have affected the precision of the estimate among whites only. While the present study collects data on OPR misuse, it does not collect information on whether participants were actually prescribed the OPR in a medical setting. Furthermore, we are unable to ascertain whether a medical provider who discriminated against study participants was likely to prescribe OPRs, or any other medications. There are limitations to the measure of perceived discrimination in epidemiological studies. These measures capture discrimination as perceived and reported by participants, which could possibly result in subjective misreporting of discrimination based on factors related to the participants’ experiences in healthcare. By focusing on individual-level experiences of discrimination, the perceived measure of discrimination may be missing macro-level sources of discrimination (institutional, policy, etc.). While study participants may be aware of discrimination happening at these levels, it is not captured in our current measure of discrimination. Our discrimination measure was treated as a binary yes/no question, and thus does not consider that some experiences of discrimination may be more severe than others. However, since there are currently no gold standard measures for capturing discrimination in epidemiological studies we believe using the perceived discrimination measure makes an important contribution to the literature despite these limitations. A final consideration is that the CARDIA cohort does not include persons from certain geographic regions of the United States or persons at high risk for substance misuse, and as such our findings regarding the experiences of discrimination and their effect on OPR misuse may not be generalizable to other study populations. In spite of these limitations, the results of this study do provide an indication that this type of interpersonal discrimination impacts misuse of OPRs, in a way that is similar to the documented relationships between perceived discrimination, mental health outcomes, and other substances of abuse [26–33].

Our study has several strengths that contribute to the current literature. The CARDIA study is one of few cohort studies with questions, repeated over time, on both discrimination in a medical care setting and OPR misuse. Although there is no objective or gold standard measure of discrimination, the experiences of discrimination instrument (EOD) used in this analysis has been previously validated, making this sample well suited for this research question[14]. We performed several sensitivity analyses and our results were unchanged, supporting the conclusions of our analyses. To account for the test/ retest validity of our discrimination measures we performed two sensitivity analyses using different definitions. In one analysis, we excluded participants who flipped their answer to the experience of discrimination between the 1992 and 2000 exam years, and results were unchanged. In another, we restricted the analysis to participants who experienced recent discrimination instead of lifetime discrimination–though we did not have the sample size to detect significance, the point estimates in our final model were similar to our main analysis. Finally, the CARDIA study is a cohort study with data from the 1990s to the present, so we were able to establish a temporal sequence between our predictor (discrimination up to year 2000) and our outcomes (OPR misuse after 2000), which was the time with the largest increase in OPR misuse in the history of the United States.

To our knowledge, there is currently no literature on whether discrimination in a medical care setting impacts OPR misuse, and the United States is amid an OPR misuse epidemic. Our findings were consistent with other studies that have found that whites report OPR misuse at higher rates than blacks[1, 5, 6, 21, 35]. Our study further suggests that discrimination increases a person’s risk of OPR misuse for both black and white persons rather than being protective against OPR misuse for blacks, and thus did not explain the black–white disparity. Our objective in this manuscript was not to identify risk factors for OPR misuse specifically, but rather to posit a possible explanation for racial disparities in OPR misuse. However, given that discriminatory treatment in a medical care setting (or any other setting) is never acceptable, in this study we have identified discrimination in a medical setting as a potential modifiable target for alleviating OPR misuse, acknowledging that other research is necessary to fully inform the prevention of OPR misuse. Further research is needed to investigate the complex mechanisms behind the black–white disparity in OPR misuse, including the source of OPR medications.

## Supporting information

S1 Table Relationship of race, discrimination in medical settings, and OPR misuse using causal mediation methods, restricted to participants whose answer to lifetime discrimination was consistent across Years 1992 and 2000, CARDIA study (N = 3,401). Model 1 is adjusted for race; Model 2 is adjusted for race, medical discrimination and confounders (parental SES, age, sex, and study site); Model 3 is adjusted for race, medical discrimination, confounders (parental SES, age, sex, and study site), and confounders/mediators (education, income, depressive symptoms, and insurance status); Model 4 is adjusted for race and medical discrimination, and uses stabilized inverse probability weights to account for the confounders and confounders/mediators (see supplemental SAS code).
(DOCX)

S2 Table Relationship of race, *recent* discrimination in medical settings, and OPR misuse using causal mediation methods, CARDIA study (N = 3,528). Recent discrimination was constructed by restricting to participants answering “Yes” to Lifetime Discrimination in 2000 but “No” in 1992. Model 1 is adjusted for race; Model 2 is adjusted for race, medical discrimination and confounders (parental SES, age, sex, and study site); Model 3 is adjusted for race, medical discrimination, confounders (parental SES, age, sex, and study site), and confounders/mediators (education, income, depressive symptoms, and insurance status); Model 4 is adjusted for race and medical discrimination, and uses stabilized inverse probability weights to account for the confounders and confounders/mediators (see supplemental SAS code).
(DOCX)

S3 Table Relationship of race, discrimination in medical settings, and OPR misuse using causal mediation methods, additionally adjusted for self-reported pain in models 3 and 4 (N = 3,528). Model 1 is adjusted for race; Model 2 is adjusted for race, medical discrimination and confounders (parental SES, age, sex, and study site); Model 3 is adjusted for race, medical discrimination, confounders (parental SES, age, sex, and study site), and confounders/mediators (education, income, depressive symptoms, insurance status and self-reported pain); Model 4 is adjusted for race and medical discrimination, and uses stabilized inverse probability weights to account for the confounders and confounders/mediators including self-reported pain.
(DOCX)

S1 File Supplemental SAS code. (DOCX)
