# Supplementary material for: Racial discrimination in medical care settings and opioid pain reliever misuse in a U.S. cohort: 1992 to 2015
Source: PLoS One. 2019 Dec 20;14(12):e0226490. doi: 10.1371/journal.pone.0226490 (PMC6924655; doi:10.1371/journal.pone.0226490)
Supplement: S1 Table — Model 1 is adjusted for race; Model 2 is adjusted for race, medical discrimination and confounders (parental SES, age, sex, and study site); Model 3 is adjusted for race, medical discrimination, confounders (parental SES, age, sex, and study site), and confounders/mediators (education, income, depressive symptoms, and insurance status); Model 4 is adjusted for race and medical discrimination, and uses stabilized inverse probability weights to account for the confounders and confounders/mediators (see supplemental SAS code). (DOCX) [file pone.0226490.s001.docx]

**S1 Table. Relationship of Race, Discrimination in Medical Settings, and OPR Misuse Using Causal Mediation Methods, Restricted to Participants Whose Answer to Lifetime Discrimination was Consistent Across Years 1992 and 2000, CARDIA Study (N=3,401).** Model 1 is adjusted for race; Model 2 is adjusted for race, medical discrimination and confounders (parental SES, age, sex, and study site); Model 3 is adjusted for race, medical discrimination, confounders (parental SES, age, sex, and study site), and confounders/mediators (education, income, depressive symptoms, and insurance status); Model 4 is adjusted for race and medical discrimination, and uses stabilized inverse probability weights to account for the confounders and confounders/mediators (see supplemental SAS code).

**Supplementary Table 1:** Relationship of Race, Discrimination in Medical Settings, and OPR Misuse Using Causal Mediation Methods, Restricted to Participants Whose Answer to Lifetime Discrimination was Consistent Across Years 1992 and 2000, CARDIA Study (N=3,401)

|  | Total effect (model 1) | | CDE: Adjusted for discrimination and confounders  (model 2) | | CDE: Adjusted for discrimination, confounders, and confounders/mediators (model 3) | | | Marginal structural model  (model 4) | |
| --- | --- | --- | --- | --- | --- | --- | --- | --- | --- |
|  | OR | 95% CI | OR | 95% CI | | OR | 95% CI | OR | 95% CI |
| Black vs. white | **0.71** | **(0.55, 0.93)** | **0.64** | **(0.45, 0.9)** | | **0.54** | **(0.37, 0.79)** | **0.65** | **(0.46, 0.91)** |
|  |  |  |  |  | |  |  |  |  |
| No discrimination vs. discrimination |  |  | **0.61** | **(0.34, 1.01)** | | **0.52** | **(0.28, 0.95)** | **0.52** | **(0.29, 0.95)** |

Model 1 is adjusted for race; Model 2 is adjusted for race, medical discrimination and confounders (parental SES, age, sex, and study site); Model 3 is adjusted for race, medical discrimination, confounders (parental SES, age, sex, and study site), and confounders/mediators (education, income, depressive symptoms, and insurance status); Model 4 is adjusted for race and medical discrimination, and uses stabilized inverse probability weights to account for the confounders and confounders/mediators (see supplemental SAS code).
