# Supplementary material for: Racial discrimination in medical care settings and opioid pain reliever misuse in a U.S. cohort: 1992 to 2015
Source: PLoS One. 2019 Dec 20;14(12):e0226490. doi: 10.1371/journal.pone.0226490 (PMC6924655; doi:10.1371/journal.pone.0226490)
Supplement: S2 Table — Recent discrimination was constructed by restricting to participants answering “Yes” to Lifetime Discrimination in 2000 but “No” in 1992. Model 1 is adjusted for race; Model 2 is adjusted for race, medical discrimination and confounders (parental SES, age, sex, and study site); Model 3 is adjusted for race, medical discrimination, confounders (parental SES, age, sex, and study site), and confounders/mediators (education, income, depressive symptoms, and insurance status); Model 4 is adjusted for race and medical discrimination, and uses stabilized inverse probability weights to account for the confounders and confounders/mediators (see supplemental SAS code). (DOCX) [file pone.0226490.s002.docx]

**S2 Table. Relationship of Race, *Recent* Discrimination in Medical Settings, and OPR Misuse Using Causal Mediation Methods, CARDIA Study (N=3,528).** Recent discrimination was constructed by restricting to participants answering “Yes” to Lifetime Discrimination in 2000 but “No” in 1992. Model 1 is adjusted for race; Model 2 is adjusted for race, medical discrimination and confounders (parental SES, age, sex, and study site); Model 3 is adjusted for race, medical discrimination, confounders (parental SES, age, sex, and study site), and confounders/mediators (education, income, depressive symptoms, and insurance status); Model 4 is adjusted for race and medical discrimination, and uses stabilized inverse probability weights to account for the confounders and confounders/mediators (see supplemental SAS code).

**Supplementary Table 2:** Relationship of Race, *Recent* Discrimination in Medical Settings, and OPR Misuse Using Causal Mediation Methods, CARDIA Study (N=3,528)

|  | Total effect (model 1) | | CDE: Adjusted for discrimination and confounders  (model 2) | | CDE: Adjusted for discrimination, confounders, and confounders/mediators (model 3) | | | Marginal structural model  (model 4) | |
| --- | --- | --- | --- | --- | --- | --- | --- | --- | --- |
|  | OR | 95% CI | OR | 95% CI | | OR | 95% CI | OR | 95% CI |
| Black vs. white | **0.71** | **(0.55, 0.93)** | **0.70** | **(0.51, 0.98)** | | **0.61** | **(0.43, 0.88)** | **0.72** | **(0.52, 0.98)** |
|  |  |  |  |  | |  |  |  |  |
| No discrimination vs. discrimination |  |  | **0.77** | **(0.35, 1.72)** | | **0.66** | **(0.29, 1.51)** | **0.66** | **(0.29, 1.49)** |

Recent discrimination was constructed by restricting to participants answering “Yes” to Lifetime Discrimination in 2000 but “No” in 1992.

Model 1 is adjusted for race; Model 2 is adjusted for race, medical discrimination and confounders (parental SES, age, sex, and study site); Model 3 is adjusted for race, medical discrimination, confounders (parental SES, age, sex, and study site), and confounders/mediators (education, income, depressive symptoms, and insurance status); Model 4 is adjusted for race and medical discrimination, and uses stabilized inverse probability weights to account for the confounders and confounders/mediators (see supplemental SAS code).
