# Supplementary material for: Racial discrimination in medical care settings and opioid pain reliever misuse in a U.S. cohort: 1992 to 2015
Source: PLoS One. 2019 Dec 20;14(12):e0226490. doi: 10.1371/journal.pone.0226490 (PMC6924655; doi:10.1371/journal.pone.0226490)
Supplement: S3 Table — Model 1 is adjusted for race; Model 2 is adjusted for race, medical discrimination and confounders (parental SES, age, sex, and study site); Model 3 is adjusted for race, medical discrimination, confounders (parental SES, age, sex, and study site), and confounders/mediators (education, income, depressive symptoms, insurance status and self-reported pain); Model 4 is adjusted for race and medical discrimination, and uses stabilized inverse probability weights to account for the confounders and confounders/mediators including self-reported pain. (DOCX) [file pone.0226490.s003.docx]

**S3 Table. Relationship of Race, Discrimination in Medical Settings, and OPR Misuse Using Causal Mediation Methods, Additionally adjusted for Self-Reported Pain in Models 3 and 4 (N= 3,528).** Model 1 is adjusted for race; Model 2 is adjusted for race, medical discrimination and confounders (parental SES, age, sex, and study site); Model 3 is adjusted for race, medical discrimination, confounders (parental SES, age, sex, and study site), and confounders/mediators (education, income, depressive symptoms, insurance status and self-reported pain); Model 4 is adjusted for race and medical discrimination, and uses stabilized inverse probability weights to account for the confounders and confounders/mediators including self-reported pain.

**Supplementary Table 3:** Relationship of Race, Discrimination in Medical Settings, and OPR Misuse Using Causal Mediation Methods, Additionally adjusted for Self-Reported Pain in Models 3 and 4 (N= 3,528)

|  | Total effect (model 1) | | CDE: Adjusted for discrimination and confounders  (model 2) | | CDE: Adjusted for discrimination, confounders, and confounders/mediators (model 3) | | | Marginal structural model  (model 4) | |
| --- | --- | --- | --- | --- | --- | --- | --- | --- | --- |
|  | OR | 95% CI | OR | 95% CI | | OR | 95% CI | OR | 95% CI |
| Black vs. white | **0.71** | **(0.55, 0.93)** | **0.63** | **(0.45, 0.9)** | | **0.56** | **(0.38, 0.84)** | **0.63** | **(0.44, 0.90)** |
|  |  |  |  |  | |  |  |  |  |
| No discrimination vs. discrimination |  |  | **0.54** | **(0.34, 0.86)** | | **0.53** | **(0.31, 0.90)** | **0.52** | **(0.31, 0.88)** |

Model 1 is adjusted for race; Model 2 is adjusted for race, medical discrimination and confounders (parental SES, age, sex, and study site); Model 3 is adjusted for race, medical discrimination, confounders (parental SES, age, sex, and study site), and confounders/mediators (education, income, depressive symptoms, insurance status and self-reported pain); Model 4 is adjusted for race and medical discrimination, and uses stabilized inverse probability weights to account for the confounders and confounders/mediators including self-reported pain.
