# Supplementary material for: Racial discrimination in medical care settings and opioid pain reliever misuse in a U.S. cohort: 1992 to 2015
Source: PLoS One. 2019 Dec 20;14(12):e0226490. doi: 10.1371/journal.pone.0226490 (PMC6924655; doi:10.1371/journal.pone.0226490)
Supplement: S1 File — (DOCX) [file pone.0226490.s004.docx]

**S1 File. Supplemental SAS Code**

**Supplemental File 1. SAS Code**

This code creates the stabilized weights, *W^M^*, to account for the measured confounding of the relation between discrimination and OPR misuse:

/* Model for calculating the numerator of weight *W^M^* */

**proc** **logistic** data=analysis descending;

ods exclude ClassLevelInfo Type3 Association FitStatistics GlobalTests Oddsratios;

class binaryRaceDiscr;

model binaryRaceDiscr = isWhite;

output out=est_prob_n (keep= ID NP_M_disc) p=NP_M_disc;

**run**;

**proc** **sort** data=est_prob_n; by ID; **run**;

/* Model for calculating the denominator of weight *W^M^**/

**proc** **logistic** data= analysis descending;

ods exclude ClassLevelInfo Type3 Association FitStatistics GlobalTests;

class isWhite isFemale binaryRaceDiscr CENTER insuranceD Ccesd16;

model binaryRaceDiscr = isWhite isFemale Age3 Center High_Par_Ed EDUC4 incomeDScale Ccesd16 insuranceD ;

output out= est_prob_d p= DP_M_Disc;

**run**;

**proc** **sort** data=est_prob_d; by ID; **run**;

/*Merge numerator and Denominator datasets*/

**data** analysis_sw;

merge est_prob_d est_prob_n ;

by ID;

/*Estimation of the stabilized IPW*/

if binaryRaceDiscr=**1** then sw_a= NP_M_disc / DP_M_Disc; /*Treated*/

else if binaryRaceDiscr=**0** then sw_a= (**1**-NP_M_disc) / (**1**-DP_M_Disc); /*Untreated*/

**run**;
